# Telehealth-Delivered Cognitive Behavioral Therapy for Insomnia in Individuals with Multiple Sclerosis: A Pilot Study

**DOI:** 10.1155/2022/7110582

**Published:** 2022-03-02

**Authors:** David Turkowitch, Rebecca Ludwig, Eryen Nelson, Michelle Drerup, Catherine F. Siengsukon

**Affiliations:** ^1^Department of Physical Therapy, Rehabilitation Science, And Athletic Training, University of Kansas Medical Center, Kansas City, KS, USA; ^2^Sleep Disorders Clinic, Cleveland Clinic, Cleveland, OH, USA

## Abstract

**Background:**

Over 50% of individuals with multiple sclerosis (MS) have moderate or severe sleep disturbances, insomnia being the most common. In-person cognitive behavioral therapy for insomnia (F2F-CBTi) is currently the first-line treatment for insomnia. However, given potential limitations to access including mobility difficulty, fatigue, or living in a rural area, telehealth-delivered CBT-I (tele-CBTi) has been considered as an alternative treatment. The purpose of this study was to assess the feasibility and treatment effect of tele-CBTi in people with MS and compare it to outcomes from a F2F-CBTi study in individuals with MS.

**Methods:**

11 individuals with MS and symptoms of insomnia participated in 6 weekly CBT-I sessions with a trained CBT-I provider via live video. Insomnia severity (ISI), sleep quality (PSQI), and fatigue severity (FSS and MFIS) were assessed pre- and posttreatment as primary outcomes. Sleep onset latency (SOL), sleep efficiency (SE) and total sleep time (TST) from the PSQI, depression (PHQ-9), anxiety (GAD-7), sleep self-efficacy (SSES), and quality of life (MSIS-29) were also assessed pre- and posttreatment as secondary outcomes.

**Results:**

Participants resided in 9 different states. Retention and adherence rates were 100%. There were significant improvements in ISI, PSQI, MFIS, FSS, SOL, SSES, PHQ-9, and MSIS-29, but not SE, TST, or GAD-7. There were no significant differences between the F2F-CBTi group and tele-CBTi group for magnitude of change in the primary outcomes (ISI, PSQI, MFIS, and FSS) or the secondary outcomes (SOL, SE, TST, SSES, PHQ-9, GAD-7, and MSIS-29).

**Conclusions:**

Tele-CBTi is feasible and has outcome measures that are similar to that of in-person CBT-I treatment. Tele-CBTi may increase access to insomnia treatment in individuals with MS.

## 1. Introduction

Multiple sclerosis (MS) is an autoimmune disease of the central nervous system characterized by demyelination and subsequent axonal degeneration that affects 1/1000 individuals in the United States [1, 2]. Common MS symptoms include fatigue, numbness, weakness, visual impairment, loss of balance, dizziness, urinary bladder urgency, and depression [2]. Also, over 50% of individuals with MS have moderate or severe sleep disturbances, with insomnia being the most common sleep disorder [3–5]. Sleep disturbances in individuals with MS have been associated with a number of symptoms including poorer cognitive performance, lower quality of life, higher disability, and increased prevalence of pain, fatigue, depression, anxiety, and sexual and bladder dysfunction [4, 6–11].

Cognitive behavioral therapy for insomnia (CBT-I) is an effective treatment for insomnia and is the recommended nonpharmacological treatment for chronic insomnia [12]. CBT-I addresses the behaviors and negative cognitions that are associated with poor sleep outcomes. CBT-I has been shown to be more effective and durable compared to pharmacological interventions, showing improved sleep outcomes persisting for up to 10 years post-CBT-I treatment [13, 14]. While CBT-I is typically delivered in-person (F2F-CBTi), one-on-one or in a group setting, there is mounting evidence that telehealth-delivered CBT-I (tele-CBTi) is an effective intervention to improve sleep outcomes in individuals with insomnia [15]. Tele-CBTi generally uses the same principles and content as F2F-CBTi, including stimulus control, time in bed restriction, sleep hygiene education, cognitive strategies, and relaxation techniques; however, it involves the use of real-time electronic video communication to administer treatment from a trained practitioner [16]. Tele-CBTi has been demonstrated to be comparably effective and has similar attrition rates as traditional in-person treatment [17].

There is emerging evidence that CBT-I is an effective treatment in people with MS. One recent study reported a significant improvement in insomnia symptoms, sleep quality, fatigue, sleep self-efficacy, and depression symptoms in individuals with MS who received CBT-I [18]. However, access to F2F-CBTi treatment is limited, particularly in rural areas, where there are not as many clinicians trained in providing CBT-I [19]. Furthermore, individuals with MS may have additional barriers to attending in-person CBT-I such as mobility restrictions and other comorbidities [10]. Another recently published study found a web-based CBT-I program tailored specifically for individuals with MS resulted in improvements in insomnia severity, sleep quality, sleep self-efficacy, and anxiety [20]. However, web-delivered CBT-I is an automated program that cannot be tailored to the specific needs of the individual. Also, the web-based CBT-I study in people with MS had an attrition rate of 50%, suggesting that acceptability of a web-delivered program is limited [20]. Tele-CBTi would allow greater accessibility of care while also including tailored treatment with a practitioner. Thus, the purpose of this study was to assess the feasibility and treatment effect of tele-CBTi in people with MS and compare it to prior F2F-CBTi outcomes in patients with MS [18].

## 2. Methods

Participants were recruited through the National Multiple Sclerosis Society's (NMSS's) website and newsletters. Individuals who had participated in prior studies or expressed interest in participating in studies were also recruited. This study was conducted April-December 2020 in accordance with the University of Kansas Medical Center's Institutional Review Board [#00142464], and informed consent was obtained. This clinical trial was not listed on ClinicalTrials.gov. Inclusion criteria included are as follows: (1) diagnosis of relapsing-remitting MS, (2) 18-80 years old, (3) ≤4 on the Patient Determined Disease Steps (PDDS) [21], (4) ≥10 or greater on the Insomnia Severity Index (ISI) [22], (5) self-reported understanding of written and spoken English, (6) access to internet service, and (7) self-reported completion of a high school diploma or equivalent. Exclusion criteria included are as follows: (1) known untreated sleep disorder, (2) >3 on the STOP BANG [23], (3) increased risk of restless legs syndrome on the Restless Legs Syndrome–Diagnostic RLS-Diagnosis Index [24], (4) ≥15 on the had a Patient Health Questionnaire-9 (PHQ-9) or endorsement of any suicidal ideation [25], (5) self-report having a nervous system disorder other than MS, (6) relapse and/or corticosteroid use in the past 8 weeks, or (7) currently performing shift work.

Feasibility was assessed by the following: (1) number of people enrolled out of the number of people contacted (recruitment), (2) number of participants who completed the study (retention), (3) number of individuals who dropped out of the study (attrition), (4) number of CBT-I sessions attended (adherence), and (5) the number of states participants reside (scope). To assess the treatment effect on insomnia symptoms (primary outcome) and sleep quality and fatigue, the Insomnia Severity Index (ISI) [22], Pittsburgh Sleep Quality Index (PSQI) [26], Fatigue Severity Scale (FSS) [27], and Modified Fatigue Impact Scale (MFIS) [28] were collected pre- and postintervention using the REDCap (Research Electronic Data Capture) [29] tool hosted at KUMC. To assess the treatment effect on the secondary outcomes of interest (sleep onset latency (SOL), sleep efficiency (SE), total sleep time (TST), depression, anxiety, sleep self-efficacy, and quality of life), SOL, SE, and TST from the PSQI [26], the Patient Health Questionnaire (PHQ-9) [25], Generalized Anxiety Disorder Assessment (GAD-7) [30], Sleep Self-Efficacy Scale (SSES) [31], and the Multiple Sclerosis Impact Scale (MSIS-29) [32] were also gathered pre- and postintervention.

All participants participated in the tele-CBTi program which consisted of 1x/week CBT-I sessions with a trained CBT-I provider for 6 weeks. The standardized CBT-I program was based on the manual by Perlis et al. and has been described previously [18, 33]. Sessions were delivered via Zoom using a HIPAA-compliant license.

Using G∗Power 3 (Heinrich Heine University Düsseldorf), we determined 10 participants were needed to detect a large effect size (*d*) of 0.9 in a two-dependent mean model for 80% power and allowing for a type 1 error of 0.05.

Data were analyzed using SPSS Statistics for Windows, version 22.0 (IBM Corp). Feasibility (recruitment, retention, attrition, adherence, and satisfaction) was assessed using frequency analysis. Change from baseline to reassessment was assessed using paired samples *t*-test. Magnitude of change was assessed using within-group effect sizes (Cohen's *d*) which were interpreted as small, *d* = 0.2; medium, *d* = 0.5; and large, *d* = 0.8. The number and percentage of participants who met the minimal clinically important difference (MCID) for the primary outcome measures were also reported.

Change scores were calculated (reassessment score–baseline score) for each primary and secondary outcome. One-way analyses of variance (ANOVAs) and Fisher's exact test analyses were used to assess for differences between the F2F-CBTi group and the tele-CBTi group in demographic variables and performance on baseline assessments at baseline [18]. One-way ANOVAs or ANCOVAs (baseline outcome included as covariate if statistically significant difference at baseline; FSS and MSIS-29 were significantly different at baseline so included as a covariate) were used to assess between-group differences in change scores [18].

## 3. Results

**Figure 1:**
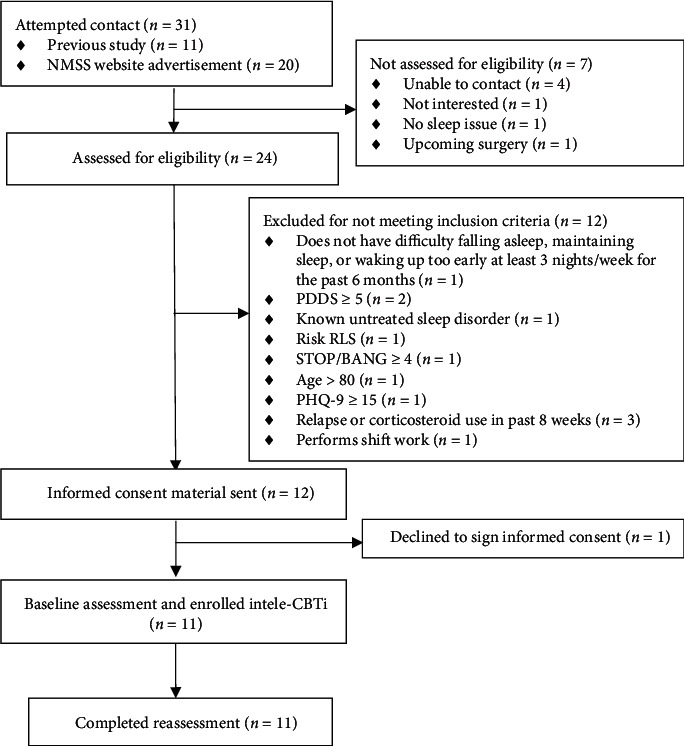
Study design.

Nine women and two men participated in the study (Table 1). The average age was 50.2 years old (SD 13.5), and average PDDS was 2.7 (SD 1.2). Participants resided in nine different states. Attempts were made to contact 31 individuals, and 11 of those individuals enrolled in the study for a recruitment rate of 35% (Figure 1). All individuals who enrolled in the study completed for a retention rate of 100% (0% attrition). All individuals attended six out of six tele-CBTi sessions for an adherence rate of 100%.

The tele-CBTi group had a significant improvement in ISI, PSQI, MFIS, FSS, SOL, SSES, PHQ-9, and MSIS-29, but not SE, TST, or GAD-7 (Table 2). Ten of the 11 participants had a MCID of at least 6 points on the ISI [34], at least 3 points on the PSQI [35], and at least ≥10 points on the MFIS [36].

There were no significant differences between the F2F-CBTi group and tele-CBTi group for sex, MS type, age, disease severity, marital, working, or smoking status, consumption of alcohol, or highest degree earned (Table 1). There were no significant differences between the F2F-CBTi group and tele-CBTi group for magnitude of change in the primary outcome measures of ISI, PSQI, MFIS, or FSS or the secondary outcome measures of SOL, SE, TST, SSES, PHQ-9, GAD-7, or MSIS-29 (Table 3).

## 4. Discussion

This was the first study that assessed the feasibility and treatment effect of tele-CBTi in individuals with MS and compared these outcomes with in-person delivery of CBT-I. This study demonstrated that tele-CBTi is feasible and produces large changes in insomnia severity, sleep quality, fatigue, sleep onset latency, sleep self-efficacy, depression, and quality of life. Furthermore, the improvements in sleep outcomes following tele-CBTi appear similar to that of F2F-CBTi suggesting that both may be effective for improving sleep outcomes, fatigue, and comorbid symptoms in people with MS.

The results that tele-CBTi improves sleep outcomes support recent studies that have shown that the principles of F2F-CBTi have been delivered effectively through different modalities, such as web-based and tele-CBTi [15, 17, 37]. The results of our study are also consistent with previous studies that found that CBT-I improves comorbid symptoms including fatigue, depression, and quality of life in people with MS specifically, as well as within the general population [15, 18, 20, 38–41].

The lack of statistically significant difference in the improvements in sleep outcomes and comorbid outcomes between the tele-CBTi and in-person CBT-I group suggests that both may be effective methods of CBT-I delivery. However, this suggestion should be viewed with caution due to the small sample size, lack of randomization between the two groups, and the study not being prospectively designed as a noninferiority study; however, these results do support the need for future studies to determine noninferiority between these two delivery methods. There has been only one study to our knowledge that has directly compared CBT-I treatment delivered in-person and via telemedicine, and they found that tele-CBTi yielded similar improvements in insomnia severity and daytime functioning as in-person CBT-I [17]. In addition, Arnedt et al. also reported that the treatment effects for both groups were maintained at 3 months posttreatment [17].

It is interesting that the tele-CBTi group had about a one-point larger effect size improvement for both fatigue scales and sleep self-efficacy than the in-person CBT-I group. Perhaps participating in a teledelivered program is less fatiguing than needing to travel and attend an in-person program. Also, it is possible that sleep self-efficacy was better enhanced because the provider was able to view the participant's home and bedroom to better tailor recommendations. Future studies are needed to support these suppositions.

In this study, tele-CBTi was administered to individuals in 9 different states, whereas the in-person CBT-I study was limited to two adjacent states. This alludes to the possible extension of access to treatment that tele-CBTi may provide, which is a significant potential benefit of tele-CBTi as there is a known paucity of trained CBT-I providers, particularly in rural areas; even many urban areas, considering the size of their populations, do not have a sufficient number of CBT-I providers [19]. Tele-CBTi may serve as a treatment option that increases access for patients while maintaining similar outcomes as that of in-person CBT-I. However, even with telemedicine extending access to CBT-I, there remains a need for more trained CBT-I providers to adequately serve those requiring CBT-I in addition to providers trained to administer tele-CBTi and infrastructure and technical support necessary for tele-CBTi.

In addition to tele-CBTi being advantageous in circumstances when there is limited provider availability, tele-CBTi also remains advantageous when disability and/or fatigue limits travel ability. Another benefit of tele-CBTi is the participant can share views of the bedroom and home environment so the provider can make recommendations for environmental modifications. On the other hand, tele-CBTi may have limitations such as the requirement of computer and internet access as well as the participant's and provider's comfort levels with technology. However, since 2000, the percent of people in the US that use the internet has been increasing; currently, 93% of the people in the US use the internet, and 85% of Americans are online daily [42, 43]. Regardless, it does seem ideal to offer in-person and tele-CBTi options if possible to allow for individual choice and considering the individual's needs. Stepped-care CBT-I treatment has been a suggested approach to allow for a “stepping” in intensity or rigor of treatment type (sleep promotion education to web-based CBT-I to tele-CBTi or face-to face CBT-I) [44, 45]. Future research is needed to identify characteristics of those who would benefit from a stepped-care approach compared to those who need tele- or in-person CBT-I initially.

A limitation of this study is the small sample size which limits the interpretability of the results; however, this study identifies the effect size of tele-CBTi in people with MS to adequately power a future randomized clinical trial to verify the efficacy of tele-CBTi in this population. Another limitation is tele-CBTi treatment was conducted during the COVID-19 pandemic whereas in-person CBT-I treatment was conducted prior to the COVID-19 pandemic so it is difficult to determine if the pandemic influenced the results. However, the fact that there were significant improvements in sleep outcomes with tele-CBTi during the pandemic further supports the utility of this delivery method. Another limitation is that the participants were not randomized into the two comparison groups so we cannot be certain that possible covariates were allocated between the two groups. However, there were no statistical differences between the two groups in demographic characteristics that may have influenced outcomes (such as age and disability). Furthermore, this study excluded individuals with severe depression and advanced disability from participating; thus, our findings are not broadly generalizable to the patient population of all people with MS.

In conclusion, tele-CBTi is feasible and improves sleep outcomes and comorbid symptoms in individuals with MS. Furthermore, there were no significant differences between the in-person and tele-CBTi groups for any of the primary or secondary outcomes measured suggesting that both delivery methods may be efficacious in people with MS. The next step is for an adequately powered randomized clinical trial to determine the efficacy of both delivery methods. Additionally, future study is warranted to determine the characteristics of individuals that benefit from each delivery method as well as from a stepped-care approach. This information would allow for low-cost, low-risk, and streamlined treatment to guide clinicians and patients towards a suitable insomnia intervention.

## Figures and Tables

**Table 1:** Descriptive statistics of the participants. Data reported as mean (standard deviation) or number (*n*). RR: relapsing-remitting; SP: secondary progressive; PDDS: Patient Determined Disease Steps.

Group	Tele-CBTi (*n* = 11)	F2F-CBTi (*n* = 10)	*p* value
Sex			
Male	2	1	1.00
Female	9	9	
Age (years)	50.3 (13.5)	51.1 (7.9)	.867
MS type			
RR	11	8	.214
SP	0	2	
PDDS	2.3 (1.2)	1.3 (2.2)	.219
Marital status (*n*)			.318
Married	7	6	
Divorced	2	2	
Single	1	2	
Other	1	0	
Working status			.128
Working	4	7	
Retired	3	0	
Unemployed	0	1	
Other	4	2	
Currently smoker			1.00
Yes	0	0	
No	11	10	
Consume alcohol			.086
Yes	3	7	
No	8	3	
Highest degree earned			.300
High school	1	4	
Associate degree	1	0	
Bachelor's degree	6	3	
Graduate degree	3	3	
State of residence			
AZ	2		
MN	1		
MO	1		
NC	2		
NJ	1		
NM	1		
SC	1		
TX	1		
WY	1		

**Table 2:** Primary and secondary outcomes at baseline (pre), reassessment (post), change score, and effect size. Data reported as mean (standard deviation). ISI: Insomnia Severity Index; PSQI: Pittsburgh Sleep Quality Index; MFIS: Modified Fatigue Impact Scale; FSS: Fatigue Severity Scale; SSES: Sleep Self-Efficacy Scale; SOL: sleep onset latency; SE: sleep efficiency; TST: total sleep time; PHQ-9: Patient Health Questionnaire, 9 items; GAD-7: Generalized Anxiety Disorder Assessment, 7 items; MSIS-29: Multiple Sclerosis Impact Scale, 29 items; ES: effect size.

Tele-CBTi					
	Pre	Post	Change	*p*	ES
*Primary outcomes*					
ISI	17.1 (3.3)	6.3 (3.3)	-10.8 (3.8)	<.001	2.815
PSQI	13.1 (2.3)	6.0 (3.8)	-7.1 (3.5)	<.001	2.006
MFIS	44.2 (15.7)	15.3 (10.2)	-28.9 (13.1)	<.001	2.193
FSS	49.5 (8.6)	26.5 (15.4)	-23.0 (16.1)	.001	1.432
*Secondary outcomes*					
SOL (min)	57.8 (29.7)	22.7 (23.7)	-35.1 (18.8)	<.001	1.959
SE (%)	64.8 (19.2)	78.3 (24.1)	13.5 (25.8)	.112	0.531
TST (min)	308.2 (71.1)	375.3 (120.2)	67.1 (103.1)	.056	0.715
SSES	21.5 (4.1)	36.4 (5.4)	14.9 (7.2)	<.001	2.077
PHQ-9	8.3 (4.2)	3.1 (3.2)	-5.2 (4.6)	.004	1.136
GAD-7	5.4 (4.6)	3.2 (3.2)	-2.2 (5.3)	.200	0.415
MSIS-29	73.8 (16.9)	50.5 (12.4)	-23.4 (18.4)	.002	1.265

**Table 3:** Primary and secondary outcomes at baseline (pre), reassessment (post), change score, and effect size. Data reported as mean (standard deviation). ISI: Insomnia Severity Index; PSQI: Pittsburgh Sleep Quality Index; MFIS: Modified Fatigue Impact Scale; FSS: Fatigue Severity Scale; SSES: Sleep Self-Efficacy Scale; SOL: sleep onset latency; SE: sleep efficiency; TST: total sleep time; PHQ-9: Patient Health Questionnaire, 9 items; GAD-7: Generalized Anxiety Disorder Assessment, 7 items; MSIS-29: Multiple Sclerosis Impact Scale, 29 items; ES: effect size.

Tele-CBTi				F2F-CBTi			
	Change	*p*	ES	Change	*p*	ES	*p*
*Primary outcomes*							
ISI	-10.8 (3.8)	<.001	2.815	-13.3 (4.9)	<.001	2.729	.210
PSQI	-7.1 (3.5)	<.001	2.006	-6.7 (2.9)	<.001	2.314	.785
MFIS	-28.9 (13.1)	<.001	2.193	-19.3 (18.2)	<.001	1.064	.178
FSS	-23.0 (16.1)	.001	1.432	-8.5 (16.2)	.043	0.524	.908
*Secondary outcomes*							
SOL	-35.1 (18.8)	<.001	1.959	-33.3 (39.2)	.025	1.117	.893
SE	13.5 (25.8)	.112	0.531	17.7 (8.8)	<.001	2.075	.635
TST	67.1 (103.1)	.056	0.715	73.0 (47.2)	.001	1.572	.870
SSES	14.9 (7.2)	<.001	2.077	11.2 (9.3)	<.001	1.221	.317
PHQ-9	-5.2 (4.6)	.004	1.136	-4.7 (5.9)	<.001	0.798	.836
GAD-7	-2.2 (5.3)	.200	0.415	-1.9 (3.4)	.049	0.549	.888
MSIS-29	-23.4 (18.4)	.002	1.265	-10.7 (11.2)	.015	0.955	.870

## Data Availability

The data that support the findings of this study are available from the corresponding author, CS, upon reasonable request.
